# The Role of Visual Performance in Fine Motor Skills

**DOI:** 10.3390/life14111354

**Published:** 2024-10-23

**Authors:** Pilar Granados-Delgado, Miriam Casares-López, Francesco Martino, Rosario González Anera, José Juan Castro-Torres

**Affiliations:** Laboratory of Vision Sciences and Applications, Department of Optics, Faculty of Sciences, University of Granada, Avenida Fuentenueva s/n, 18071 Granada, Spain; pilargrd@ugr.es (P.G.-D.); clmiriam@ugr.es (M.C.-L.); francesco@ugr.es (F.M.); rganera@ugr.es (R.G.A.)

**Keywords:** visual performance, visual acuity, contrast sensitivity, disability glare, fine motor skills

## Abstract

The aim of this study was to analyse the relationship between fine motor skills (FMSs) and visual performance. Thirty young participants with normal binocular vision performed five fine motor tasks: Purdue, Grooved, and O’Connor pegboards, a needle threading task, and a water pouring task, which were characterised by the time taken to complete the task, the number of pegs inserted, the error made in pouring the water, and the volume spilled. To evaluate visual performance, near visual acuity, near contrast sensitivity (CS), and disability glare were assessed. Fine motor skills and visual performance were assessed under monocular and binocular viewing conditions. An overall visual performance score (OVPS) and an overall fine motor skills score (OFMSS) were calculated. All visual functions measured binocularly were better than in monocular conditions, and all FMSs tasks were performed worse monocularly than binocularly (*p* < 0.001), except for the error made in the water pouring task (*p* = 0.024). There was a positive correlation between OVPS and OFMSS (rho = 0.329; *p* = 0.010). The regression model showed that the OFMSS can be predicted by age and CS at 21.3%. Individuals with normal binocular vision and better near visual function exhibit superior fine motor abilities. CS stands out as the visual function that has the greatest bearing on the performance of FMSs.

## 1. Introduction

The visual system provides information about the objects in our environment through the interpretation of the images received by the eyes and subsequently processed by the brain. In daily activities, humans interact with objects using their hands. Thus, interactions between our visual system and hand movements have been studied in many disciplines, including optometry. In terms of vision, there are different metrics for quantifying visual performance. These include visual acuity (VA), contrast sensitivity (CS), and disability glare [1,2,3]. Contrast sensitivity (CS) is the ability to perceive sharp and clear outlines of very small objects. The term used to describe the visual impairment that occurs in the presence of bright light sources is “disability glare” [1]. Sometimes the loss of CS can be more disturbing than the loss of visual acuity because the real world is not constantly in high level of contrast. Additionally, it is well known that the human binocular visual system has the ability to fuse two images into a single image and to perceive depth through the disparity that exists between those two images. The loss of binocular vision negatively impacts the daily life of sufferers [4].

Visuomotor coordination is a hallmark of human evolution and a key aspect of manual dexterity [5], which is involved in several everyday tasks, such as sewing, cooking, or doing a puzzle. The manual dexterity factor is defined as the ability to make skilful, controlled arm–hand manipulations of larger objects [6] and the finger dexterity factor is defined as the ability to make rapid, skilful, controlled manipulative movements of small objects, primarily involving the fingers [7]. Thus, the literature suggests that there are multiple types of dexterity. According to factor analysis findings, pegboard dexterity and finger tapping assess different dimensions of manual dexterity. For this reason, there are several methods for analysing and evaluating manual dexterity and, within this, fine motor skills (FMSs). This dexterity can be quantified as the time required to complete a defined task, like pouring a fluid into a recipient or threading a needle, but it could also be the time needed to complete a pegboard test, such as the O’Connor test or Grooved pegboard test [8,9,10,11]. Most studies on this topic focus on examining the precise position of the hands during tasks involving FMSs, as well as assessing certain visual functions [12,13,14,15]. On the other hand, several studies have demonstrated that visual impairment affects the performance of tasks involving manual dexterity [12,16,17] as well as everyday tasks such as driving [18,19,20], thus showing the importance of binocular vision. Previous studies have used and compared different standardised manual and finger dexterity tests in populations with and without mental or physical conditions [10,21,22,23]. However, they did not assess the influence of vision on manual dexterity. Other studies found an association between visual performance and manual tasks simulating everyday tasks involving grasping [4,24,25], showing that the presence of some degree of binocular vision is beneficial for the performance of certain sensorimotor tasks [15]. Other authors monitored a bead threading task using a motion capture system, finding that a greater maximum reach velocity was linked to improved vergence function. Reduced stereoacuity thresholds correlated with a decrease in grip duration, and improved accommodative function was linked to a shorter duration of positioning [14].

In addition to the aforementioned binocular vision, some studies have demonstrated that CS and disability glare (DG) are associated with better performance in tasks that require an accurate visual function, such as driving [26,27]. Changes in visual quality (considering different visual functions) and their effect on binocular vision and driving performance have also been investigated. It has been observed that the greater the interocular differences, the lower the binocular summation of the visual functions (VA and CS) and the poorer the stereoacuity [28]. Therefore, we hypothesize that manual dexterity will be impaired when developing daily activities in monocular vision without the full benefit of binocular vision functions. In view of the above, we also hypothesize that visual quality, specifically contrast sensitivity, may have a relationship with the performance of manual dexterity tasks, particularly those involving FMSs, and which are visually demanding.

Thus, the aim of this study was to determine to what degree vision (including VA, CS, and binocular vision), particularly near vision, is involved in the performance of manual tasks involving FMSs, using five different tests and tasks performed by participants with normal binocular vision.

## 2. Materials and Methods

### 2.1. Subjects

A total of 30 subjects were enrolled in the study (10 males and 20 females) with a mean age of 24.7 ± 4.2 years. The inclusion criteria were age between 18 and 40 years (non-presbyopic), corrected binocular VA of at least 1.0 (decimal notation) for near and distance vision, normal binocular vision (fusion and stereopsis), fusional vergences within normal values, and no asthenopia. Additionally, the refractive history was evaluated, and participants with high astigmatism, amblyopia, or a history of treatment for visual abnormalities were excluded. The mean (±SD) spherical refractive error was −2.61 ± 3.03 D and the mean astigmatism was −0.74 ± 0.54 D, ranging from 0.00 D to −2.50 D. All the participants signed a written informed consent form in accordance with the Declaration of Helsinki [29]. The procedures described were approved by the Human Research Ethics Committee of the University of Granada (1256/CEIH/2020).

A thorough visual examination was carried out prior to the first measurement session to ensure that the participants had their best corrected visual acuity (BCVA) and balanced binocular vision. Stereopsis was evaluated using the Randot Stereotest (Stereo Optical, Inc., Chicago, IL, USA) at near vision (40 cm) to verify that there was good binocular integrity. The average stereoacuity for the participants was 28.5 ± 12.4 arcsec, which is within the normal range (<40 arcsec) for correct binocular vision [30,31,32,33].

In addition, they had to complete two questionnaires to confirm that they had normal vision and no asthenopia: the visual discomfort scale (CONLON) and the Binocular Vision Discomfort Questionnaire (BVDQ). The CONLON questionnaire uses percentages: a total score of 24 points or less means that the participants can be classified in the low visual discomfort group [34]. The BVDQ does not have a standardised score; however, a lower score is associated with less discomfort [35]. For the CONLON and BVDQ questionnaires, we obtained average scores of 12.5 ± 8.1 points and 13.6 ± 8.6 points, respectively, confirming that our sample did not experience visual discomfort.

### 2.2. Visual Performance

Visual performance was assessed by means of different visual functions, including visual acuity, contrast sensitivity, and disability glare.

#### 2.2.1. Visual Acuity

Near and distance VA was assessed in monocular and binocular viewing conditions using the OptoTab VA screening test (SmarThings4Vision, Zaragoza, Spain), a test used in previous studies [36]. The test was performed for near (0.5 m) and distance (5.5 m) vision. The software employs optotypes to identify wrong answers. It provides an exact calculation of the decimal visual acuity (VA).

#### 2.2.2. Contrast Sensitivity

Contrast sensitivity (CS) was measured at 0.5 m using the OptoTab contrast sensitivity test (SmarThings4Vision), which comprises sinusoidal grids showing different orientations. Five spatial frequencies were assessed: 1.5, 3, 6, 12, and 18 cycles per degree (cpd) of visual angle. A total of nine contrast levels were evaluated for each spatial frequency as follows: the observers had to recognize and indicate whether the grid was inclined to the right, the left, or vertical; if they responded correctly, the contrast level decreased until they were no longer able to correctly indicate the inclination of the grating, reaching the contrast threshold for that condition and reporting the corresponding CS value. In order to establish correlations between this visual function and FMSs, the CS for each subject and condition was reported as the average of the CS for all of the spatial frequencies tested, as other works have done [28].

#### 2.2.3. Disability Glare

**Figure 1:**
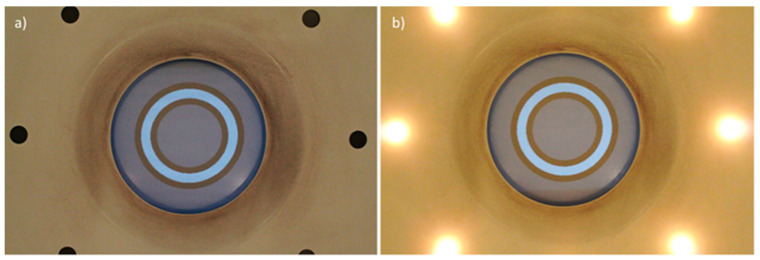
Central light ring used as a visual stimulus without (**a**) and with (**b**) the presence of glare in the Takagi CGT-1000 glare tester. Six of the eight LEDs composing the glare source can be observed.

Disability glare (DG) is the loss of retinal image contrast due to intraocular light scattering or straylight, which is caused by imperfections and loss of transparency of the optical media [37]. This value was obtained by evaluating CS at six target sizes (6.3, 4, 2.5, 1.6, 1, and 0.7 degrees, corresponding to spatial frequencies of 1.0, 1.7, 2.6, 4.2, 6.6, and 10.4 cpd, respectively) with and without the presence of glare using the CGT-1000 device (Takagi, Japan) [38]. The instrument evaluates 12-step contrast thresholds using a visual stimulus consisting of a central luminous ring. The glare source is composed of 8 LEDs distributed around the contrast stimulus (Figure 1). This device measures the monocular contrast threshold, which was determined by the lowest contrast level perceived by each participant. For each stimulus size, the DG was reported as the difference between the contrast threshold and the contrast threshold with the presence of glare, so that the higher the DG value, the greater the glare experienced by the subject and the poorer the visual performance in the presence of light sources [39]. For each subject and condition, the DG was reported as the DG averaged for all spatial frequencies (target sizes). Each subject was tested under monocular and binocular conditions with a modification of the instrument that enabled both eyes to be assessed simultaneously.

To obtain a single metric for visual function, the Overall Visual Performance Score (OVPS) was calculated. This score was obtained as the averaged z-scores obtained from each of the individual variables: VA, mean CS and DG. For each subject, the z-score of a variable is defined as the number of standard deviations below (negative) or above (positive) the group mean. The scores of DG were converted so that positive z-scores represented a better performance than the mean.

### 2.3. Fine Motor Skills

**Figure 2:**
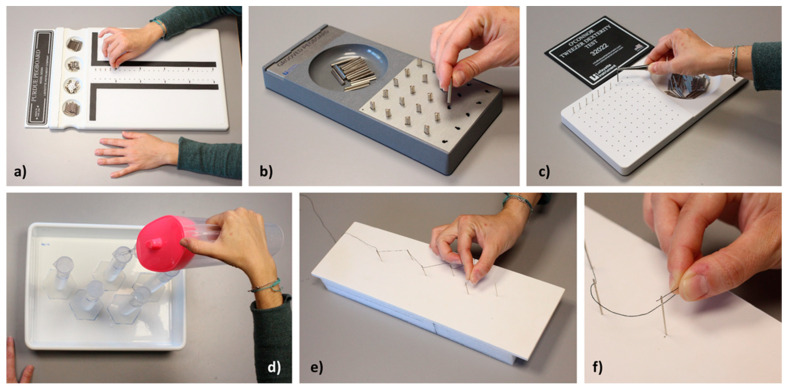
(**a**) Purdue Pegboard; (**b**) Grooved Pegboard; (**c**) O´Connor Pegboard; (**d**) water jug and test tubes to be used in the water pouring task; (**e**,**f**) sewing thread, needles and threading device for the threading task.

A total of 5 different tests were performed to assess FMSs under three viewing conditions: monocularly (right and left eye) and binocularly (Figure 2). The task order and viewing conditions were randomised. The participants were seated in a chair 50 cm in front of the table. Training was performed for each test prior to the final measurement to minimise learning and fatigue effects. The tests were performed under photopic conditions. The illuminance on the test board was measured using a PCE-170A light meter (PCE Instruments, PCE Deutschland GmbH, Meschede, Germany), obtaining a mean illuminance of 753 ± 120 lux (measured in 9 points with the lux meter placed directly above the test board). The following FMSs tests were performed:

#### 2.3.1. Purdue Pegboard (Lafayette Instruments, Lafayette, IN, USA)

This is a standardised test used to assess unimanual and bimanual motor skills [40,41,42]. The board presents two parallel vertical rows of 25 holes each (Figure 2a). Pegs are located in the extreme right-hand and left-hand wells at the top of the board, while collars and washers are put in the two middle wells. The main task in the test consists of placing as many pegs or pieces (pegs, collars, and washers) as possible into a row of holes on the board, from top to bottom, within a given time. Four different tasks (subtests) were performed with this test. Three of the subtests consisted of inserting as many pegs as possible (1) into the holes of the dominant-hand row using the dominant hand (first subtest), (2) into the non-dominant-hand row using the non-dominant hand (second subtest), and (3) into both rows using both hands simultaneously (third subtest). In these subtests, the number of pegs inserted in 30 s was recorded. In the fourth subtest, the participants used both hands alternately to construct assemblies in the dominant-hand row of the board. Each assembly included a total of four pieces: a peg, then a washer over it, then a collar, and then another washer. The subject had to complete as many assemblies as possible within 60 s. In this subtest, the number of pieces placed correctly was recorded.

#### 2.3.2. Grooved Pegboard (Lafayette Instruments, Lafayette, IN, USA)

This is used to measure hand–eye coordination and motor speed. This is a strong predictor of motor performance in healthy adults [43]. The test board comprises a perforated board with 25 holes with the same asymmetrical profile but in different orientations, and a well containing the pegs (Figure 2b). The pegs have a protruding section that matches the shape of the holes so the pegs must be inserted so that the orientation of its section coincides with that of the hole. The participants had to use their dominant hand to insert the pegs row by row from left to right (right-handed), or in the opposite direction (right to left) if they were left-handed. The time taken to place all the pegs (in seconds) was measured using a stopwatch.

#### 2.3.3. O’Connor Tweezer Dexterity Test (Lafayette Instruments, Lafayette, IN, USA)

This consists of a board with 100 holes and a well containing pegs (Figure 2c). This test measures the speed with which a subject is able to pick up pegs and insert them into the holes using a pair of tweezers [11]. In our experiment, the subject had to insert the pegs in the holes of the first row (one peg per hole, 10 holes in total) in the shortest possible time while holding the tweezers with the dominant hand. The time taken to place the pegs in the first row (in seconds) was recorded.

#### 2.3.4. Water Pouring Task

This manual dexterity task consisted of pouring 450 mL of water into five test tubes (diameter 2.7 cm and capacity 100 mL) arranged in a zigzag pattern on a tray (Figure 2d). A similar test has been used in previous research [15]. Each test tube was marked at 90 mL. The participants had to hold a jug (containing 600 mL of water) with their dominant hand and fill the five test tubes, from right to left for right-handed participants (left to right for left-handed participants), up to the mark. We quantified the time taken to complete the task (in seconds), the error made in filling each tube (in mL), and the water spilled onto the tray (in mL).

#### 2.3.5. Threading Task

In this task, the participants were asked to thread six needles with a black thread over a white background in the shortest possible time, using their dominant hand (Figure 2e,f). The participants were given specific instructions in order to avoid any tactile reference when performing the tasks. The time taken to thread the first four needles (hole size 1.3 × 8.0 mm) was recorded, as was the time taken to thread all of them. The last two needles presented a higher level of difficulty since the needle eye was smaller (1.0 × 4.0 mm).

To obtain an overall measure of FMSs, the Overall Fine Motor Skills Score (OFMSS) was calculated in the same way as described for the OVPS. This score was obtained as the averaged z-scores for each of the individual FMSs parameter variables for each participant. The scores were converted so that the more positive the score, the better the FMSs performance.

### 2.4. Statistical Analysis

Sample size was determined by power calculation using G*Power v.3.1.9.2 software. The analysis indicated that 21 participants were needed to achieve equivalent effect sizes (Cohen d [0.8–1.2]) with 95% power on all of the key measures in this study (vision and FMSs variables).

Statistical analyses were performed using the software SPSS 28.0 (SPSS Inc., Chicago, IL, USA). To analyse the relationship between FMSs performance and the visual parameters (near VA, near CS and DG), an eye was randomly selected and the means and standard deviations were calculated for each visual parameter and the FMSs metric. Prior to the data analysis, the Kolmogorov–Smirnov test was run on all variables (vision and tasks). Wilcoxson’s Z-test was applied to determine if there were statistically significant differences between the monocular and binocular conditions. The relationship between visual function and FMSs was first explored with a bivariate correlation analysis. A Spearman correlation analysis was conducted when normality could not be assumed, and a Pearson correlation analysis was performed when the data were normally distributed. Finally, to determine how visual function and other factors predict the performance of FMSs tasks, a regression model (with a forward stepwise selection) was run with the OFMSS as a dependent variable, visual variables (DG, CS and VA) and age as covariates, and the total CONLON score as a fixed factor. The significance level was set at *p* < 0.05 for all tests.

## 3. Results

### 3.1. Visual Performance and FMSs

No biological-sex-related differences were found for visual function and the performance of FMSs (*p* < 0.05). The results from the visual assessment (by means of near visual acuity, contrast sensitivity, and disability glare) are summarised in Table 1. In the case of contrast sensitivity and disability glare, both the total average values and the average values for each of the spatial frequencies or angular sizes are shown. We obtained better results for VA and DG in the binocular condition, but there were no statistically significant differences between the two viewing conditions. However, significant results were obtained from the detailed analysis of DG when comparing the two conditions, specifically for the angle sizes of 6.3, 4, 2.5, and 1.6 degrees. Statistically significant differences were observed in CS measured under binocular conditions versus monocular measurements, with better contrast sensitivities obtained in binocular conditions (*p* < 0.05). The average CS for each spatial frequency measured is also shown in Table 1. A better CS was obtained in binocular conditions for all spatial frequencies except for 6 cpd (*p* > 0.05).

Table 2 shows the results for the FMSs. The scores corresponding to the binocular viewing conditions obtained from the three validated tests (Purdue, O’Connor, and Grooved) are consistent with both the standardised scores provided by each manufacturer and by previous studies [22,40,44]. The time modification when performing the adapted O’Connor Tweezer Dexterity task was considered.

The tasks for which the time required to complete them was assessed were performed significantly faster under binocular viewing conditions compared to monocular viewing (*p* < 0.05). When the task was performed under binocular viewing, less water was spilled (*p* < 0.05) and there was, on average, greater accuracy when filling the test tubes, although the differences were not significant (*p* > 0.05). Finally, performing the Purdue pegboard task binocularly resulted in more pegs inserted in 30 s for all conditions (dominant and non-dominant hand, and both hands) and more assemblies made within 60 s (*p* < 0.05). In addition, in the Purdue pegboard test, there were significant differences between the results using the dominant and non-dominant hand. The results were better when performing the peg insertion with the dominant hand (Z= −5.864; *p* < 0.001).

The correlation between the three standardised tests (Purdue, Grooved, and O’Connor) and the two tests that simulated everyday tasks (needle threading and water pouring) was also analysed. We obtained a significant correlation (rho = 0.412; *p* = 0.024), showing that the better the three standardised dexterity tests were performed, the better the simulated everyday tasks were performed.

### 3.2. Correlations Between Vision and FMSs

Only the following variables were normally distributed: Grooved test performance time, pieces inserted in the Purdue test assembly task, O’Connor test performance time, and water pouring time. We used Spearman’s correlation index for our analysis as most of the variables were not normally distributed.

**Figure 3:**
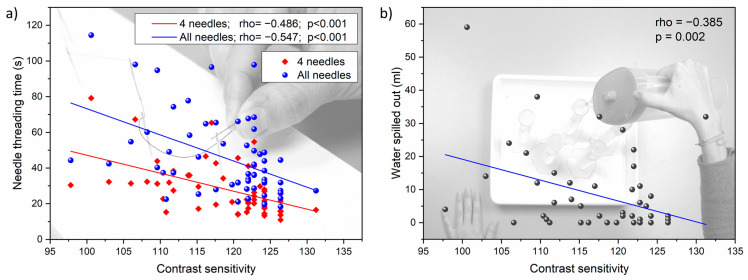
(**a**) Scatter plot showing the relationship between needle threading time (in seconds) and mean near CS for the two levels of difficulty (threading of 4 and 6 needles); (**b**) Scatter plot showing the relationship between water spilled (in mL) during the pouring task and mean near CS.

Figure 3 shows the parameters of the two daily visual tasks as a function of the averaged contrast sensitivity. We found significant descending correlations between the mean CS and the two FMSs tasks: the threading task for the two different levels of difficulty (rho = −0.547, *p* < 0.001; and rho = −0.486, *p* < 0.001, respectively) (Figure 3a), and the water spilled during the pouring task (rho = −0.385, *p* = 0.002) (Figure 3b). Each point represents the mean CS for each participant in each viewing visual condition (monocular and binocular) (abscissas), and their corresponding values of time spent to thread the needles (s) or amount of spilled water (mL) (ordinates in Figure 3a,b, respectively). The results indicated that the better the CS, the less time needed to complete the threading task and the lower the volume of water spilled.

Significant correlations between the FMSs tasks and the spatial frequencies of the CS are shown in Table 3. The results indicate that CS correlates positively with the number of pegs inserted in the Purdue pegboard task, suggesting that better CS at the highest spatial frequency (18 cpd) is associated with a higher number of pegs inserted.

Significant negative correlations were found for the remaining tasks, indicating that as the CS improved, less time was needed to complete the FMSs tasks. Additionally, for the two tasks that simulate everyday tasks (water pouring and threading), there were significant correlations, especially at the low frequencies (1.5 and 3 cpd).

Table 4 reports the significant correlations obtained between DG and FMSs for each quantified stimulus size. These results revealed an association between the DG values obtained for each stimulus angle size in the contrast threshold test, with and without glare, and the performance of the manual tasks. Elevated DG values suggest a greater deterioration of CS under glare conditions. Overall, the findings demonstrated that greater impairment from glare leads to poorer performance of FMSs tasks. Specifically, more time was required to complete the Purdue pegboard tasks and fewer pieces were introduced when CS deteriorated. Significant correlations were found for almost all tasks for the 6.3 and 4-degree stimulus sizes; however, an association was observed for the 2.5-degree stimulus size only for the needle threading and water-pouring tasks. Correlations were found between DG for the 1.6-degree stimulus size and the Purdue pegboard tasks. DG was greater at this size and was associated with more water being spilled during the water-pouring task. For the smallest size, no correlations were found.

**Figure 4:**
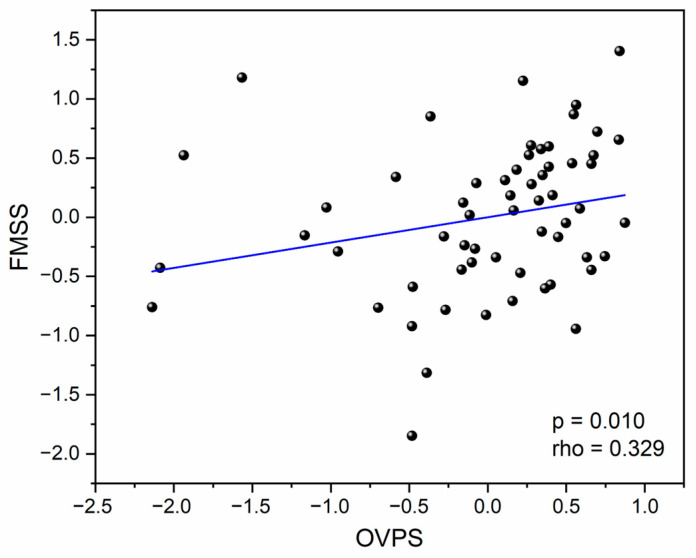
The overall fine motor skills score (OFMSS) as a function of the overall visual performance score (OVPS).

To analyse the relationship between visual performance and FMSs, first, the OVPS and OFMSS indices were calculated. Figure 4 shows the correlation between these two indices (rho = 0.329; *p*-value = 0.010). The higher the OVPS, the better the visual performance. Likewise, the higher the OFMSS, the better the performance of FMSs. This positive correlation suggests that individuals with better near visual function (in terms of VA, CS, and DG) exhibit superior fine motor abilities.

To find the best linear model to predict the FMSs score as a function of visual parameters and other variables, a multiple linear regression analysis was run with the OFMSS as the dependent variable and the different visual functions and age as covariates. The visual parameter included was the near CS. This forward stepwise method selected the age of the participants. The results of this analysis are shown in Table 5.

According to the R-squared value obtained, this model explains 21.3% of the *OFMSS*. The *OFMSS* would be given by the following regression line Equation (1)

(1) $$OFMSS=0.038\times CS+0.042\times age\left(years\right)-5.497$$

The standard deviations were 0.010, 0.017, and 1.322, for the first, second, and third terms of the equation, respectively.

The *OFMSS*, i.e., the performance of FMSs, can therefore be predicted by this linear regression model at 21.3%. The predictive factors include age and the average *CS* measured for near vision, with *CS* being the most important contributor to the regression model.

## 4. Discussion

In this study, we investigated the relationship between visual function in healthy non-presbyopic adults, assessed by means of visual acuity (VA), contrast sensitivity (CS) and disability glare (DG), as well as fine motor skills (FMSs). We analysed adults instead of children because the cortical maturation of visual, somatosensory, and motor processing provides the neural substrate for refining FMSs in adolescence [5]. Firstly, our results from the clinical tests of visual function showed better outcomes for binocular viewing, in line with previous studies [24,26,45,46,47].

To evaluate FMSs, we analysed the performance of five different tasks: three standardised dexterity tests (Purdue, Grooved and O’Connor tests) and two everyday fine-motor tasks. The first three tests have been described in the literature as tests that allow us to measure motor function. In particular, factor analysis studies have shown that the Purdue pegboard test is supported by a finger dexterity factor [7]. Meanwhile, the needle threading and the water pouring tests enabled us to characterise two everyday fine-motor tasks performed using the dominant hand, similar to that reported in a previous work [4]. In our research, we obtained a correlation between the two types of tests (standardised dexterity tests and daily tasks). These results may indicate that these two types of tests can be used to reliably assess FMSs. However, further research into this issue is needed to reach a solid conclusion.

The improvement when performing FMSs binocularly is in line with previous studies [9,16,17,48]. These results suggest that the contribution of depth vision and binocular summation is reflected in improved performance in all tasks related to controlling the terminal reach and grasp. According to Melmoth and Grant [12], these benefits derive from binocular disparity processing linked to changes in relative hand–pegboard distance, and this depth information is independently used to regulate the progress of the approaching hand and to guide the task, thereby ensuring that the grip is securely applied.

We also found differences when comparing the results of the Purdue pegboard test with the dominant and non-dominant hand. The results were better when inserting the pegs with the dominant hand, as reflected in previous studies [49,50].

As mentioned above, the main aim of our research was to investigate the relationship between VA, CS, DG, and FMSs. We focused on these three visual functions because, in clinical practice, VA is the parameter most commonly used to characterise vision, and CS defines important characteristics that are not captured by VA alone. Previous studies have shown that CS plays an important role in the performance of daily tasks that involve visuomotor skills, including driving [26], endoscopic surgery [51], and sports [52,53]. In addition, previous studies on amblyopic participants have shown that even after the total recovery of VA following refractive amblyopia treatment, interocular differences in CS were not equalised and stereopsis did not return to normal values [54]. Previous studies have shown that optical correction would improve refractive amblyopia but may nevertheless present an impairment in fine motor skills [55] or have poor visual development due, for example, to high astigmatism [56]. This aspect shows the importance of taking into consideration the refractive history of the subjects and whether they have presented any type of vision anomaly such as amblyopia. In the present study, subjects with high astigmatism, amblyopia, or a history of treatment for visual abnormalities were excluded. Furthermore, subjects included in the study showed normal values in the results of the standardized manual dexterity tests. In our study we also analysed DG, incorporating information on how CS is affected by glare. It is known that visual performance is more sensitive to glare in central vision than in peripheral vision [57]. We found decreased DG in both monocular and binocular vision because all our participants were healthy, but with significant differences for the bigger stimulus sizes.

In general, more significant correlations were obtained for the needle threading and water pouring, which represent real daily tasks that are visually demanding, particularly the needle threading task. We found that the better the CS, the less time was needed to complete the needle threading task, and the less water was spilled during the pouring task. Meanwhile, for DG, the correlations indicated that greater glare-induced disability leads to poorer performance in the FMSs tasks. These findings support our initial hypothesis, confirming the influence of CS in daily fine-motor tasks involving near vision and are in line with the previous studies discussed above [58]. In each of the five tasks, we observed varying levels of contrast that define, for example, the holes in the pegboards and the components to be retrieved and inserted, as well as the edges of the test tubes or the eyes of the needles used. This might suggest that improved CS is related to a stronger visual signal, resulting in a faster initial movement impulse.

Correlations between FMSs and CS and DG for each spatial frequency and angle size were analysed. Although correlations were found for both high and low frequencies in the case of the standardised tests, more correlations were found for low spatial frequencies in the needle threading and water pouring tasks. These findings could be explained by the fact that, although the sizes subtended by the different stimuli were smaller, the five FMSs tasks performed in our study presented a high degree of difficulty and involved spatial vision for planning and performance. CS performance appears to be a determinant in visuospatial processing [59]. Grandjean et al. suggested that the performance of CS tests is mediated, in part, by visuospatial skills, and they showed that the designs used in the test to assess CS may require different types of skills. According to our results, this seems reasonable since our FMSs tasks also involved visuospatial skills. On the other hand, the low spatial frequencies are much larger and easier for the eyes to detect, and the eyes would be most sensitive to detecting objects under low contrast objects when the spatial frequencies are around 3 to 5 cpd. In our study, the difference between monocular and binocular contrast sensitivity (under normal conditions, CS, and assessing glare, DG) is significantly higher for low and medium spatial frequencies, so these spatial frequencies could have a greater impact on the performance of some FMSs. This would explain why the DG correlates better with FMSs for lower spatial frequencies (higher angle sizes, 6.3 and 4.0 degrees) so that the higher the DG value, the worse the FMSs performance. These correlations are also observed for CS and daily tasks, further showing that the highest spatial frequency evaluated in this work (18 cpd) also plays an important role. In addition, the results provided by Grandjean et al. suggest that attention and executive system functioning may also contribute to performance in CS tests [59]. Similarly, these functions are present in our FMSs tasks. Another interesting factor to be considered is the sensory feedback from low-threshold receptors in the skin. This feedback plays a crucial role in hand control in FMSs [43].

On the other hand, we calculated the OFMSS, enabling us to group different FMSs tasks into a single metric. By incorporating all the variables that characterise these tests, we were able to jointly evaluate the different dimensions of manual dexterity involved in the FMSs, providing a more complex characterisation. The correlations are only used to identify the relationship between two variables; however, a regression analysis is required to determine the value of one variable in terms of another [60,61]. Our results indicated that the near CS and age predict the FMSs in 21.3% of cases. We found that age is a predictor of FMSs performance. Therefore, according to our results, there seems to be an age-related improvement in the performance of FMSs in adults aged 18 to 40. However, according to the literature, from the ages of 60 to 65, there is a deterioration in these abilities [62,63]. Thus, the regression model obtained in this study would only apply to young adults up to 40 years of age, and further studies would be needed to report a regression model covering a wider age range. These results may be related and supported by other findings. Recent research has shown that medium spatial frequency ranges are most optimal for testing visual function, such as 3 cpd in age macular degeneration (AMD) [64] and 4 cpd in amblyopia [56]. In line with the predictions for FMSs, measuring CS at intermediate spatial frequencies may be a good way to proceed. At the visual level, along the lines suggested previously, additional visual measures, among which we highlight CS, are necessary to understand the impact of vision and therefore of vision loss in daily life [58].

It should also be noted that we only analysed healthy participants and is important to analyse other groups of subjects, such as those with any type of non-strabismic binocular anomaly, amblyopia, or degrading visual function, using filters or lenses. It would also be interesting to study the influence of visual performance on FMSs in older subjects, who suffer from deteriorated fine motor skills but also decreased contrast sensitivity, especially at high frequencies [58].

Recent studies have monitored FMSs performance and yielded promising preliminary results using new systems [65]. The combination of these findings and the measured visual functions can offer further insight into the role of the visual system in the various motor skills. Additionally, analysing relevant visual variables alongside FMSs performance could enhance our understanding of that role.

## 5. Conclusions

Binocular vision is superior to monocular vision for the visual functions studied (visual acuity, contrast sensitivity, and disability glare) and results in better performance in the execution of various fine motor tasks. There is also a correlation between visual performance and fine motor skills, so that the better the visual performance, the better the fine motor skills. The results of this study provide important insights into the role of near contrast sensitivity in the performance of complex manipulation tasks in non-presbyopic adults. In individuals with normal binocular vision, contrast sensitivity stands out as the visual function with the greatest relevance in terms of fine motor performance. In particular, low-medium spatial frequencies correlate more closely with daily tasks in healthy adults, suggesting the implication of spatial vision in the planning and execution of these tasks. These results highlight the importance of visual functions, such as contrast sensitivity, indicating that this should be included in routine ophthalmology or optometry vision screenings. The assessment of these visual functions may also be useful in other clinical areas such as psychology or neurology, where manual dexterity or fine motor skills are often analysed.

## Figures and Tables

**Table 1:** Mean values for the visual parameters studied under the two viewing conditions.

		Viewing Condition		Statistic	
		Monocular	Binocular	Wilcoxon’s Z-Test	*p*-Values
Near VA		1.21 ± 0.05	1.23 ± 0.04	−1.934	0.053
Mean DG		0.031 ± 0.029	0.019 ± 0.012	−1.635	0.102
DG for each stimulus size	6.3 deg	0.014 ± 0.012	0.004 ± 0.004	−4.345	<0.001 *
	4 deg	0.015 ± 0.014	0.005 ± 0.006	−3.804	<0.001 *
	2.5 deg	0.014 ± 0.027	0.007 ± 0.008	−3.351	0.001 *
	1.6 deg	0.024 ± 0.013	0.017 ± 0.012	−2.437	0.015 *
	1 deg	0.038 ± 0.038	0.0279 ± 0.018	−0.772	0.440
	0.7 deg	0.080 ± 0.123	0.053 ± 0.054	−0.285	0.776
Mean CS		115.96 ± 8.05	121.98 ± 4.64	−3.469	0.001 *
CS for each spatial frequency	1.5 cpd	80.70 ± 15.66	90.60 ± 10.35	−2.412	0.016 *
	3 cpd	149.57 ± 19.58	159.40 ± 11.77	−2.434	0.015 *
	6 cpd	163.43 ± 5.54	165.63 ± 2.01	−1.897	0.058
	12 cpd	124.10 ± 14.47	129.80 ± 4.475	−2.068	0.039 *
	18 cpd	60.83 ± 19.54	64.47 ± 6.91	−1.958	0.050 *

* Significant differences. VA, visual acuity; CS, contrast sensitivity; DG, disability glare.

**Table 2:** Mean values of parameters characterizing the performance of fine motor skills in two different viewing conditions (monocular and binocular).

Test		Viewing Condition		Statistic	
		Monocular	Binocular	Wilcoxon’s Z-Test	*p*-Values
Purdue pegboard	Dominant hand (no. pegs)	13.77 ± 1.92	15.40 ± 1.65	−4.307	<0.001 *
	Non-dominant hand (no. pegs)	12.43 ± 1.65	13.90 ± 1.90	−4.081	<0.001 *
	Both hands (no. pegs)	10.03 ± 1.43	11.63 ± 1.38	−4.523	<0.001 *
	Assemblies (no. pegs)	36.30 ± 6.57	40.37 ± 6.55	−3.949	<0.001 *
O´Connor tweezer dexterity	Performance time (s)	42.16 ± 6.58	36.40 ± 7.31	−3.960	<0.001 *
Grooved pegboard	Performance time (s)	63.48 ± 8.36	58.84 ± 7.88	−3.990	<0.001 *
Needle threading	4-needle performance time (s)	37.28 ± 14.40	18.84 ± 4.71	−4.782	<0.001 *
	All-needle performance time (s)	60.07 ± 22.76	30.81 ± 8.00	−4.782	<0.001 *
Water pouring	Performance time (s)	42.31 ± 10.64	37.33 ± 8.85	−3.671	<0.001 *
	Error made (mL)	9.23 ± 7.42	7.80 ± 4.92	−1.133	0.257
	Water spilled (mL)	13.47 ± 13.63	0.93 ± 1.93	−4.408	<0.001 *

* Significant differences.

**Table 3:** Significant correlations between FMSs and contrast sensitivity at near distance, for each spatial frequency measured.

CS Spatial Frequencies (cpd)						
		1.5	3	6	12	18
Purdue pegboard	Non-dominant hand (no. pegs)					rho: 0.278 *p*-value: 0.032
	Both hands (no. pegs)					rho: 0.255 *p*-value: 0.050
O’Connor tweezer dexterity	Performance time (s)		rho: −0.307 *p*-value: 0.017			
Needle threading	4-needle performance time (s)	rho: −0.354 *p*-value: 0.005	rho: −0.382 *p*-value: 0.003		rho: −0.351 *p*-value: 0.006	rho: −0.277 *p*-value: 0.032
	All-needle performance time (s)	rho: −0.337 *p*-value: 0.009	rho: −0.326 *p*-value: 0.011		rho: −0.336 *p*-value: 0.009	rho: −0.256 *p*-value: 0.048
Water pouring	Performance time (s)		rho: −0.344 *p*-value: 0.007			
	Water spilled (mL)	rho: −0.319 *p*-value: 0.013	rho: −0.345 *p*-value: 0.007	rho: −0.265 *p*-value: 0.040		

**Table 4:** Significant correlations between FMSs and disability glare at near distance, for each stimulus angle size measured.

		Disability Glare Stimulus Angle Sizes (Degree)				
		6.3	4	2.5	1.6	1
Purdue pegboard	Dominant hand (no. pegs)	rho: −0.311; *p*-value: 0.016	rho: −0.262; *p*-value: 0.043			
	Non-dominant hand (no. pegs)		rho: −0.265; *p*-value: 0.041		rho: −0.301; *p*-value: 0.020	
	Both hands (no. pegs)	rho: −0.361; *p*-value: 0.005	rho: −0.423; *p*-value: 0.001		rho: −0.292; *p*-value: 0.024	
O’Connor tweezer dexterity	Performance time (s)	rho: 0.331; *p*-value: 0.010	rho: 0.272; *p*-value: 0.035			
Needle threading	4-needle performance time (s)	rho: 0.447; *p*-value: 0.000	rho: 0.552; *p*-value: 0.000	rho: 0.378; *p*-value: 0.003		
	All-needle performance time (s)	rho: 0.433; *p*-value: 0.001	rho: 0.517; *p*-value: 0.000	rho: 0.360; *p*-value: 0.005		
Water pouring	Performance time (s)		rho: 0.271; *p*-value: 0.036	rho: 0.298; *p*-value: 0.021		
	Water spilled (mL)	rho: 0.427; *p*-value: 0.001	rho: 0.389; *p*-value: 0.002	rho: 0.343; *p*-value: 0.007	rho: 0.371; *p*-value: 0.004	

**Table 5:** Results of the multiple linear regression analysis. The coefficients, the standard deviation (SD), the t-statistic, the *p*-values, and the confidence interval (CI) are shown.

	Coefficient	SD	t-Statistic	*p*-Value	95% CI
OFMSS	−5.497	1.322	−4.158	0.000	[−8.143, −2.850]
Mean near CS	0.038	0.010	3.672	0.001	[0.017, 0.058]
Age (years)	0.042	0.017	2.385	0.020	[0.007, 0.077]

## Data Availability

The data that support the findings of this study are available from the corresponding author upon reasonable request.
